# To the Meeting of the American Medical Association

**Published:** 1885-04

**Authors:** 


TO THE MEETING OF THE AMERICAN MEDICAL ASSOCIATION.

The next annual meeting of this body, as has been before announced in the JOURNAL, will be held in the city of New Orleans, commencing Tuesday, April 28, 1885, at 10 o'clock, A. M., and continuing four days.

A special train of Pullman cars will leave Boston on Friday, April 24th, at 3 o'clock P. M., reaching Buffalo sometime Saturday morning, April 25th. This train will be under the control of Dr. W. E. Anthony, of Providence, R. I., who is sparing no pains in his efforts to make every provision for a safe, speedy and comfortable transit for physicians and their families to the Crescent City. Meals will be furnished at appropriate points along the route, at the uniform rate of sixty cents per meal, coupons for which will be supplied on board the train. The rate of fare from Buffalo has not yet been fixed, but it will be, of course, but little under the Rochester rate, $31.65.

For further particulars regarding the matter, our readers are referred to Dr. W. W. Potter, 306 Franklin street, who is in correspondence with Dr. Anthony, and will gladly furnish any information, from time to time, which he may obtain, concerning the details of the tour.

LATER—Dr. Anthony desires the insertion of the following:

"Delegates to the American Medical Association, and others along the line of the N. Y. C. R. R. who wish to avail themselves of the privilege of the special train to New Orleans, can do so by making application to Dr. W. E. Anthony, of Providence, R. I., and remitting $11.00, the price of double berth to New Orleans, or $22.00 for the round trip, before April 15th instant. This train will run through without change. Fare for round trip (tickets good until May 31st): From Albany, $36.40; Syracuse, $33.25; Rochester, $31.65."

---

THE *Niagara Index* has reached us, giving the official correspondence between Prof. C. C. F. Gay and the Trustees of Niagara University. Dr. Gay, on account of failing health, has resigned the chair of operative and clinical surgery, and has been tendered, as a graceful acknowledgment of his ability and high standing in the profession, the position of Emeritus Professor. We regret the necessity which compelled our colleague to relinquish the active work of teaching, for which he is so well qualified by long experience and study. The action of the Trustees, however, retain him in the faculty, without the labor which an active professorship imposes.

---

THE Spring Course in the Niagara Medical College opened April 1st, to continue eight weeks, which, with the regular course, makes eight months of continuous medical instruction. The attendance of students is very good, and the advantages offered excellent. The course is largely recitative and clinical. The close of the Session of 1884-5 took place Tuesday, March 24th, all the faculty being present, and appropriate remarks were made. The graded examinations demonstrated that excellent work has been done by both teachers and pupils in this institution.

